# Upconversion Emission and Dual-Mode Sensing Characteristics of NaYF_4_:Yb^3+^/Er^3+^ Microcrystals at High and Ultralow Temperatures

**DOI:** 10.3390/nano14100871

**Published:** 2024-05-17

**Authors:** Xinyi Xu, Zhaojin Wang, Jin Hou, Tian Zhang, Xin Zhao, Siyi Di, Zijie Li

**Affiliations:** 1 Institute of Physics and Optoelectronics Technology, Baoji University of Arts and Sciences, Baoji 721016, China; xuxinyi@stu.bjwlxy.edu.cn (X.X.); houjin@stu.bjwlxy.edu.cn (J.H.); zhangtian@stu.bjwlxy.edu.cn (T.Z.); zhaoxin@stu.bjwlxy.edu.cn (X.Z.); disiyi@stu.bjwlxy.edu.cn (S.D.); lizijie@stu.bjwlxy.edu.cn (Z.L.); 2 Baoji Ultrafast Lasers and Advanced Materials Science and Technology Center, Baoji 721016, China

**Keywords:** temperature sensing, upconversion luminescence, dual mode, ultralow temperature

## Abstract

In this study, we investigate micrometer-sized NaYF_4_ crystals double-doped with Yb^3+^/Er^3+^ lanthanide ions, designed for temperature-sensing applications. In contrast to previous studies, which focused predominantly on the high-temperature regime, our investigation spans a comprehensive range of both high and ultralow temperatures. We explore the relationship between temperature and the upconversion luminescence (UCL) spectra in both frequency and time domains. Our findings highlight the strong dependence of these spectral characteristics of lanthanide-doped NaYF_4_ crystals on temperature. Furthermore, we introduce a dual-mode luminescence temperature measurement technique, leveraging the upconversion emission intensity ratio for both green and red emissions. This study also examines the correlation between temperature sensing, energy level disparities, and thermal coupling in Er^3+^ ions across various temperature scales. Our research contributes to advancing the understanding and application of lanthanide-doped materials, setting a foundation for future innovations in temperature sensing across diverse fields.

## 1. Introduction

Upconversion luminescence overcomes the limitations of shortwave excitation by offering enhanced light stability, improved safety, and reduced ultraviolet (UV) pollution. Lanthanide rare-earth-ion-doped upconversion luminescent materials exhibit stable physicochemical properties and efficient conversion optical properties. These materials are versatile and find application in various fields, including biological imaging, 3D displays, light sources, biological monitoring, and temperature sensing [1,2,3,4,5,6,7]. Sodium yttrium fluoride (NaYF_4_) is recognized for its high transmittance and low phonon energy, making it an excellent candidate for upconversion luminescence [8,9,10]. Incorporating erbium (Er^3+^) as an activator in NaYF_4_ is particularly effective owing to its numerous energy levels and prolonged metastable energy level lifetime. Additionally, the combination of ytterbium (Yb^3+^) as a sensitizer ion, characterized by its high energy transfer efficiency, plays a pivotal role in enhancing the upconversion luminescence efficiency of Er^3+^. This enhancement is particularly notable when Er^3+^ is excited by a 980 nm laser [11,12,13,14,15]. The synergistic effect of these lanthanide ions significantly improves the overall performance and effectiveness of upconversion luminescence processes in NaYF_4_ systems.

Recently, the relationship between the upconversion emission intensity and temperature has gained interest as a basis for an innovative approach to measuring temperature. For example, in a pioneering work, Feng et al. discovered that the β-NaYF_4_:Er^3+^ crystal exhibits an increased absolute sensitivity (*S_A_*), achieving a notable value of 0.1914 K^−1^ at 443 K [16]. Meng et al. employed a solvothermal method to fabricate a NaYF_4_:Yb^3+^/Tm^3+^@NaGdF_4_:Nd^3+^/Yb^3+^ system for temperature sensing [17]. Their experiments revealed that this material exhibits a maximum relative sensitivity (*S_R_*) of 0.0088 K^−1^ between 285 and 345 K and 0.0189 K^−1^ between 345 and 495 K. Recently, Yin et al. synthesized K_3_Gd(PO_4_)_2_:Yb^3+^/Er^3+^/Tm^3+^ via a solid-state reaction, achieving a maximum *S_R_* of 0.0078 K^−1^ between 300 and 675 K [18]. However, most existing research has predominantly focused on high-temperature conditions. For instance, Gao et al. synthesized a NaBi(WO_4_)_2_ phosphor material co-doped with Er^3+^/Yb^3+^ [19], achieving a maximum *S_A_* of 0.0127 K^−1^ between 298 and 373 K. This was determined based on the variation in the green upconversion emission intensity ratio (FIR) with ambient temperature. Additionally, Li et al. prepared a fluorescent film material and evaluated its temperature-sensing capabilities [20]. Focusing on the green emission peak of the film across a temperature range of 299–359 K and using the FIR method, they found a maximum sensitivity of 0.0143 K^−1^ at 299 K.

However, research on the temperature characteristics of red emission peaks in Yb^3+^/Er^3+^ dual-doped crystal systems at low temperatures remains scarce [21,22]. To fill this gap, in the present work, our study encompasses a wide range of temperatures, specifically from the high-temperature range of 353–453 K to the low/ultralow temperature range of 55–230 K. In this extended range, we investigated the correlation between temperature and the frequency- and time-domain upconversion luminescence spectra for a dual-doped NaYF_4_ system. We characterized the system through X-ray diffraction (XRD) and scanning electron microscopy (SEM). In our experiments, we recorded the variable temperature spectra of the material under both high- and low-temperature conditions using a spectrometer. Employing the FIR method, we also explored the temperature-sensing characteristics of Er^3+^. This investigation focused on two green photothermal coupling levels of Er^3+^ (^2^H_11/2_ and ^4^S_3/2_), as well as the red ^4^F_9/2(1)_ and ^4^F_9/2(2)_ emission levels. This methodology offers a thorough insight into how the system reacts to variations in temperature, by examining its behavior across various emission levels. Such a detailed examination enhances our understanding of the temperature-dependent properties of the system, thus contributing valuable knowledge to the field of temperature-sensitive luminescent materials.

## 2. Experimental Details

### 2.1. Materials and Equipment

In our experiments, we predominantly employed sodium citrate (purity 99%), NH_4_F (purity 96%), and lanthanide-doped nitrate hexahydrate (RE(NO_3_)_3_·6H_2_O, where RE represents Y, Yb, or Er) (purity 99.99%) as the key experimental reagents, among others.

The crystal structure of the sample was analyzed using XRD (Brooke D2, Hamburg, Germany). The spectrum and lifetime of the system were recorded using a transient fluorescence spectrometer (FLS-980) and 980 nm laser (Edinburgh, UK), respectively. The low-temperature upconversion luminescence spectra were recorded using a temperature controller from Oxford Instrument, Oxford, UK (Optistat Dry), and the high-temperature upconversion luminescence spectra were recorded using a temperature controller from Shanghai Tianmei Technology Co., Ltd., Shanghai, China (TCB1402C).

### 2.2. Sample Preparation

Micron-sized NaYF_4_:20%Yb^3+^/2%Er^3+^ crystals were hydrothermally synthesized. Initially, RE(NO_3_)_3_·6H_2_O was carefully weighed in the required proportions. Subsequently, sodium citrate and deionized water were added to the mixture. The mixture was blended for 20 min using a multi-headed magnetic heat mixer. Ammonium fluoride (NH_4_F) was then added with stirring until complete dissolution. Subsequently, the solution was transferred to a reaction kettle at 200 °C and allowed to dry for 24 h. Finally, the sample was washed and dried at 60 °C, thus yielding micron-sized, white NaYF_4_:20%Yb^3+^/2%Er^3+^ crystals.

## 3. Sample Characterizations

**Figure 1:**
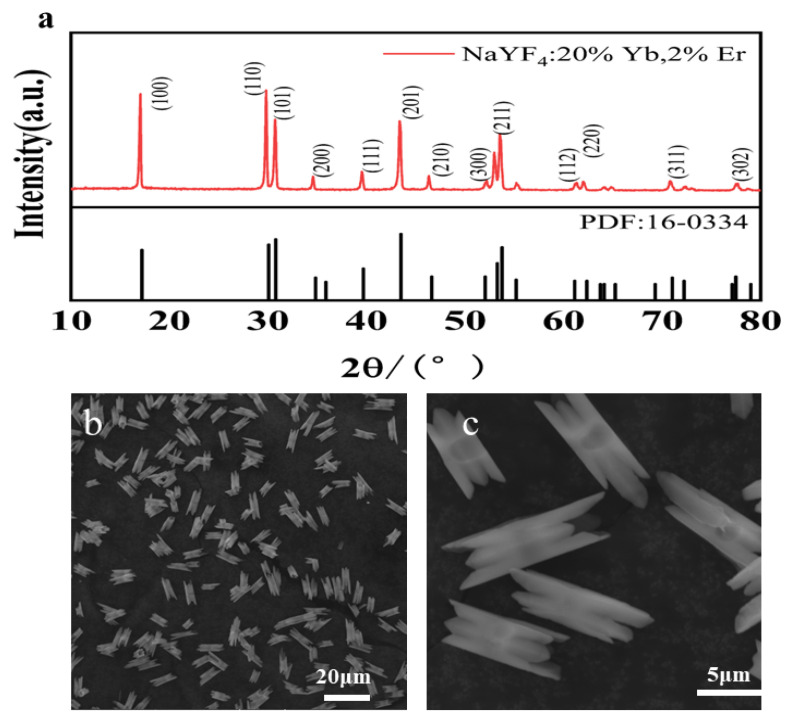
(**a**) XRD spectra; (**b**,**c**) SEM images of the system.

Figure 1a presents the XRD analysis of the crystals. The characterization plot reveals that the diffraction positions of the system closely match each peak of the standard card (PDF#16-0334), and no additional diffraction peak was observed. This indicates the successful preparation of NaYF_4_ microcrystals co-doped with Er^3+^ and Yb^3+^ in a hexagonal phase. Figure 1b,c display the SEM images of the system, illustrating evenly arranged and uniformly distributed microcrystals. These crystals show consistent morphology and size, with no clustering. Notably, a hollow cylinder, measuring approximately 12 μm in length and 3 μm in width, features smooth midsections and slightly tapered ends.

## 4. Temperature-Dependent Luminescence Characteristics

**Figure 2:**
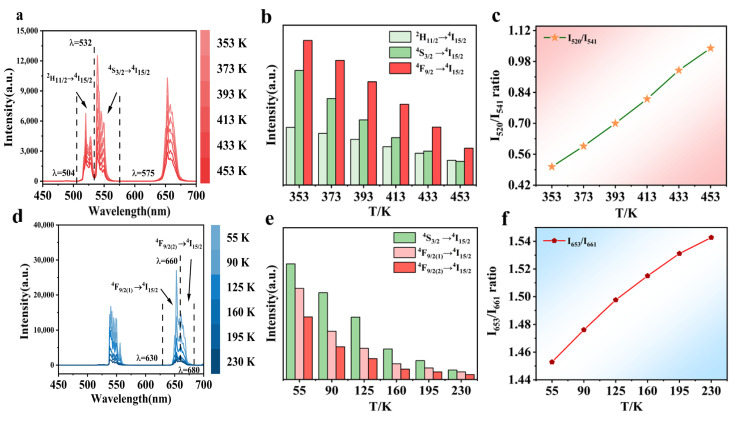
(**a**) High-temperature upconversion luminescence spectra (λ_ex_ = 980 nm at 800 mW cm^−2^); (**b**) the temperature is 353–453 K, and the integral area of different luminescent bands is shown in the histogram; (**c**) *I*_520_/*I*_541_ ratio; (**d**) low-temperature upconversion luminescence spectrum; (**e**) the temperature is 55–230 K, and the integral area of different luminescent bands is shown in the histogram; and (**f**) *I*_653_/*I*_661_ ratio.

Figure 2 shows the spectral changes in the system under the influence of temperature when the power density of 980 nm laser is 800 mW cm^−2^. The dashed line of Figure 2a,d is the dividing line between the different luminescence bands. As shown in Figure 2a, the high-temperature upconversion luminescence spectrum exhibits three primary peaks at 520, 541, and 653 nm. However, at low temperatures, the green emission peak exists only at 541 nm, as shown in Figure 2d. The green emission bands of 504–532 and 532–575 nm originate from the ^2^H_11/2_→^4^I_15/2_ and ^4^S_3/2_→^4^I_15/2_ radiation transitions, whereas the red emission bands of 630–660 and 660–680 nm originate from the ^4^F_9/2(1)_→^4^I_15/2_ and ^4^F_9/2(2)_→^4^I_15/2_ radiation transitions. Figure 2b,e present histograms with temperature plotted on the x-axis and the integral area of the emission peak on the y-axis. Within the temperature range of 353–453 K, the emission peak at 541 nm decreases sharply, whereas the upconversion emission intensity at 520 nm declines more gradually. Within the low-temperature range of 55–230 K, the emission intensity is moderate at 653 nm and more pronounced at 661 nm. Figure 2c shows a monotonically increasing trend for the ratio *I*_520_/*I*_541_ of the integral areas at 520 and 541 nm in the 353–453 K range. Figure 2f shows a monotonically increasing trend for the ratio *I*_653_/*I*_661_ of integral areas at 653 and 661 nm in the 55–230 K range.

**Figure 3:**
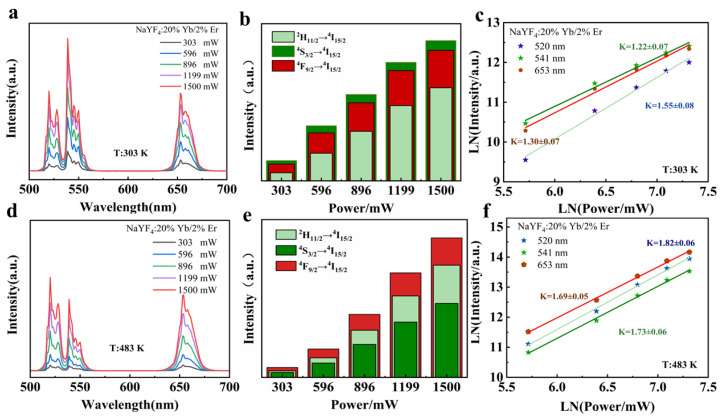
Luminescence and power relationships at different temperatures. (**a**) Luminescence spectrum at 303 K; (**b**) correlation between the integral area of upconverted emission intensity and pump power at 303 K; (**c**) relationship between the emission intensity and incident power at 303 K; (**d**) luminescence spectrum at 483 K; (**e**) correlation between the integral area of upconverted emission intensity and pump power at 483 K; and (**f**) relationship between the emission intensity and incident power at 483 K.

To understand the UC emission mechanism, we examined the luminescence spectra of the system for different power densities at 303 and 483 K upon excitation by a 980 nm laser, as shown in Figure 3a,d. At 303 K, the emission peak at 541 nm is significantly higher than that at 520 nm, and at 483 K, the spectral phenomenon is the opposite. Figure 3b,e show the correlation between the integrated areas of the emission peaks at 303 and 483 K and the different pump powers. At 303 K, the emission peak intensity is the highest at 541 nm, lower at 653 nm, and lowest at 520 nm. Conversely, at 483 K, the peak intensity is the highest at 653 nm, lower at 520 nm, and lowest at 541 nm. Figure 3c,f present the logarithmic intensity (*lnI*) and logarithmic pump power (*lnP*) plots of the system at 520, 541, and 653 nm, based on the $I\propto P^{n}\;$ characteristic [23]. The slope *n* indicates the number of low-energy photons needed for the upconversion process. At 303 K, the slopes at 520, 541, and 653 nm are 1.53, 1.22, and 1.30, respectively, whereas, at 483 K, they are 1.82, 1.73, and 1.69, respectively. This suggests that the emissions at 520, 541, and 653 nm follow a two-photon process at both 303 and 483 K [24].

**Figure 4:**
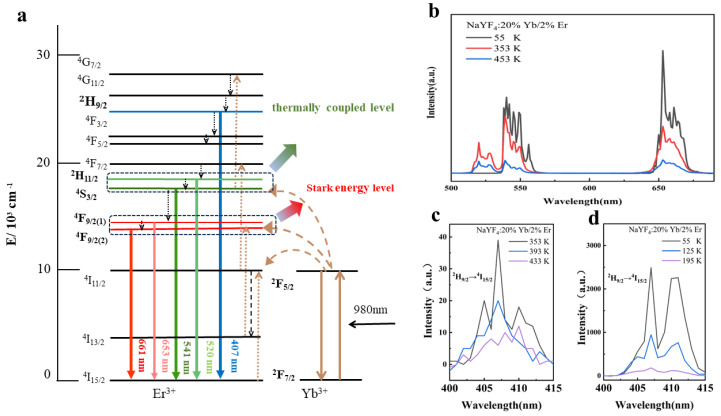
(**a**) Energy levels of Er^3+^ and Yb^3+^; (**b**) relationship between the temperature and luminous intensity; (**c**) high-temperature spectrum at 407 nm (^2^H_9/2_→^4^I_15/2_); and (**d**) low-temperature spectrum at 407 nm (^2^H_9/2_→^4^I_15/2_).

Figure 4a shows the details of the energy exchange process in a system doped with different rare-earth ions. The green arrow points to the two thermally coupled levels of Er^3+^, ^2^H_11/2_, and ^4^S_3/2_. The red arrow points to the Stark split levels of Er^3+^, ^4^F_9/2(1)_, and ^4^F_9/2(2)_. The mechanism starts with Yb^3+^ ions at the ^2^F_7/2_ level absorbing the 980 nm laser energy, thus stimulating the ^2^F_7/2_→^2^F_5/2_ transition and initially sensitizing Er^3+^ ions. These Er^3+^ ions undergo the ^4^I_15/2_→^4^I_11/2_ transition, with some undergoing the Yb^3+^-sensitized ^4^I_11/2_→^4^F_7/2_ transition. Owing to the instability of the ^4^F_7/2_ level, these ions rapidly relax to the ^2^H_11/2_ and ^4^S_3/2_ levels without radiation and then emit green light upon returning to the ground state. Another part of the particle population at the ^2^H_11/2_ level relaxes non-radiatively to ^4^S_3/2_, absorbs Yb^3+^ energy from the ^4^S_3/2_→^4^G_7/2_ transition, relaxes non-radiatively to ^2^H_9/2_, and then emits blue light (407 nm) upon returning to the ground state. This suggests that the emissions at 407 nm follow a three-photon process [25]. Meanwhile, the remaining ions undergo the ^4^I_11/2_→^4^I_13/2_ transition without radiation, absorbing energy from Yb^3+^ to reach the ^4^F_9/2_ level, and subsequently combine with ions relaxing from the ^4^S_3/2_ to ^4^F_9/2_ levels. Then, these ions emit red light as they return to the ground state [26].

As shown in Figure 3a, the emission peak at 541 nm is significantly higher than that at 520 nm; however, Figure 3d shows the opposite. As the temperature rises, the particle population increases at the higher-energy ^2^H_11/2_ level and decreases at the lower ^4^S_3/2_ level. Consequently, at 483 K, the luminescence intensity at 520 nm increases, whereas that at 541 nm decreases. Furthermore, the non-radiative relaxation probabilities of the ^4^S_3/2_→^4^F_9/2_ and ^4^I_11/2_→^4^I_13/2_ transitions increase with temperature, enhancing the ^4^I_13/2_→^4^F_9/2_ transition. Thus, the most intense luminescence at 653 nm is observed at 483 K. Figure 4c shows a decrease in the fluorescence intensity at 407 nm when the temperature is raised from 353 to 453 K, indicating that more ions transition non-radiatively from the ^2^H_9/2_ to ^2^H_11/2_ and ^4^S_3/2_ levels. This results in an increased particle population at the ^2^H_11/2_ and ^4^S_3/2_ levels. Therefore, the slope is steeper at 483 K than at 303 K, as depicted in Figure 3f.

Compared with that at high temperature, the green emission peak at low temperature can only be observed at 541 nm, whereas it is imperceptible at 520 nm, as shown in Figure 4b. This is because, at a low temperature (55 K), the population of particles at the ^2^H_11/2_ level is relatively small, with most particles populating the lower ^4^S_3/2_ level. Consequently, the luminescence at 541 nm is prominent, whereas that at 520 nm is less pronounced. According to the Boltzmann distribution law, an increase in temperature leads to enhanced lattice vibrations, an increase in particle population at higher energy levels, and a corresponding decrease in particle population at lower energy levels. Therefore, at 353 K, the particle population at the ^4^S_3/2_ level progressively increases by absorbing energy from interlattice vibrations, eventually reaching the ^2^H_11/2_ level, which leads to light emission at both 520 and 541 nm. We also observe that when the temperature surpasses 353 K, temperature quenching becomes the primary factor influencing the luminescence. As the temperature continues to rise, the probability of non-radiative multi-phonon transitions increases, leading to temperature quenching in the system. Consequently, the intensity of upconversion luminescence is reduced for both the red and green emissions. Figure 4c shows a decrease in the upconversion emission intensity at 407 nm when the temperature is raised from 353 to 453 K, which indicates that more ions transition non-radiatively from the ^2^H_9/2_ to the ^2^H_11/2_ and ^4^S_3/2_ levels. This results in an increased particle population at the ^2^H_11/2_ and ^4^S_3/2_ levels. The rising temperature increases the probability of non-radiative ^2^H_9/2_→^2^H_11/2_/^4^S_3/2_ relaxation. Figure 4d shows a decrease in the upconversion emission intensity at 407 nm when the temperature is raised from 55 to 195 K. The emission intensity at low temperatures is much greater than that at high temperatures, which suggests that the non-radiative transition is strongly inhibited at low temperatures.

**Figure 5:**
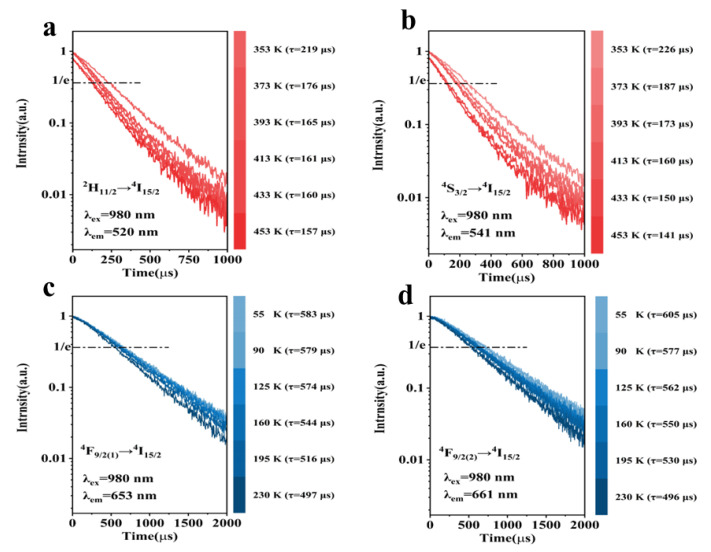
High-temperature luminescence decay kinetics at (**a**) 520 nm (^2^H_9/2_→^4^I_15/2_) and (**b**) 541 nm (^4^S_3/2_→^4^I_15/2_) and low-temperature luminescence decay kinetics at (**c**) 653 nm (^4^F_9/2(1)_→^4^I_15/2_) and (**d**) 661 nm (^4^F_9/2(2)_→^4^I_15/2_).

To further validate the luminescence performance, luminescence decay kinetics measurements were performed on the Er^3+^ emission peaks at 520, 541, 653, and 661 nm, followed by data fitting using the following exponential function [27]:

(1) $$y=y_{0}+Ae^{-t/\tau }$$

where *A* and $\;y_{0}$ are constants, and τ represents the luminescence decay kinetics. The emission peak was fitted exponentially. The luminescence decay kinetics are shown in Figure 5. Figure 5a,b reveal that as the temperature increased from 353 to 453 K, the luminescence decay kinetics at 520 nm decreased from 219 to 157 μs, and the luminescence decay kinetics at 541 nm decreased from 226 to 141 μs. The lifetime decayed slightly faster at 541 nm than at 520 nm. As shown in Figure 5c,d, when the temperature dropped from 230 to 55 K, the luminescence decay kinetics at 653 nm increased from 497 to 583 μs. Meanwhile, the luminescence decay kinetics at 661 nm increased considerably from 496 to 605 μs. Figure 2e confirms the rapid change in emission intensity at 661 nm and the moderate alteration in emission intensity at 653 nm. These observations indicate that higher temperatures correspond to shorter decay kinetics. The lifetime decay is defined as below [28]:

(2) $$\tau =\frac{1}{\Gamma_{rad}+k_{nr}}$$

where τ is the luminescence decay kinetics and $\Gamma_{rad}\;$ and $k_{nr}\;$ are the radiative and non-radiative transition probabilities, respectively. As the temperature increases, the probability of non-radiative relaxation increases, resulting in a decreasing luminescence decay kinetics. As shown in Figure 5, the lifetime changes at 541 and 661 nm are greater than those at 520 and 653 nm. With rising temperatures, particles in the lower ^4^S_3/2_ and ^4^F_9/2(2)_ energy levels transition to the higher ^2^H_11/2_ and ^4^F_9/2(1)_ levels through multi-phonon assistance, leading to a faster change in population, which results in a faster decay of lifetime. As the temperature rises, the decay kinetics decrease noticeably, indicating a correlation between the temperature and luminescence decay kinetics. This relationship can be utilized in temperature measurement applications.

## 5. Dual-Mode Luminescence Characteristics

### 5.1. Temperature-Sensing Characteristics in Dual Mode

The energy-level gap, known as the thermal coupling energy level range, ranges from 200 to 2000 cm^−1^ and conforms to the Boltzmann distribution law. For a given energy-level gap ΔE, Equation (3) can be employed to ascertain the optimal temperature ($T_{opt}$) range, as follows [29]:

(3) $$\frac{\Delta E}{\left(2+\sqrt{2}\right)k_{B}}\ll T_{opt}\ll \frac{\Delta E}{2k_{B}}$$

where $K_{B}\;$ is Boltzmann’s constant.

When the energy-level gap of 800 cm^−1^ [30] between the ^2^H_11/2_ and ^4^S_3/2_ levels in Er^3+^ is substituted into Equation (3), it yields an optimal temperature measurement range from 338 to 576 K. Similarly, a difference of 100 cm^−1^ between the ^4^F_9/2(1)_ and ^4^F_9/2(2)_ levels results in an optimal temperature range of 42–72 K. In practical materials, thermally-coupled energy levels can experience Stark effects, leading to spectral splitting. In our system, the light emission resulting from such energy-level splitting also shows a temperature-dependent relationship. However, the rate of change decreases as the energy-level gap decreases. Consequently, when the energy-level interval is small, it no longer reflects a Stark energy level but rather signifies an effective energy-level difference.

#### 5.1.1. Dual-Mode Sensing Characteristics at High Temperatures

For the thermal populations of the ^2^H_11/2_ and ^4^S_3/2_ levels in Er^3+^, the emission intensity follows the Boltzmann distribution law [31,32,33], which is expressed as below:

(4) $$FIR=\frac{I_{520}}{I_{541}}=A*\exp\left(-\frac{∆E}{K_{B}T}\right)+C$$

where *A* is a constant, *I*_520_ and *I*_541_ represent the integrated intensities, ∆*E* is the energy level gap, *T* denotes the thermodynamic temperature, and *C* is a correction factor.

**Figure 6:**
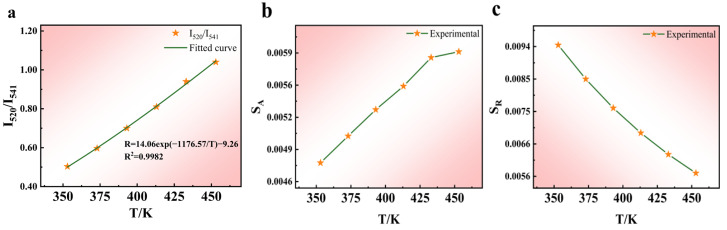
Temperature-sensing characteristics of the green part. (**a**) FIR and *T* fitting, (**b**) *S_A_* vs. *T* relationship diagram, and (**c**) *S_R_* vs. *T* relationship diagram.

Figure 6a demonstrates that the ratio of *I*_520_*/I*_541_ fitting increases with temperature. The energy difference (∆*E*) between the ^2^H_11/2_ and ^4^S_3/2_ levels is 800 cm^−1^, which corresponds to the thermally coupled energy level. According to the Boltzmann distribution law, higher temperatures lead to stronger lattice vibrations, which facilitates the electron transition from the ^4^S_3/2_ state to the high-energy ^2^H_11/2_ level. This is achieved by absorbing interlattice vibration energy to establish a quasi-thermal equilibrium. The increase in the radiative ^2^H_11/2_→^4^I_15/2_ transition probability leads to enhanced luminescence at 520 nm and reduced luminescence at 541 nm. By fitting the experimental data according to Equation (4), ∆*E*/*k* was calculated to be 1176.57 K^−1^, resulting in an experimental ∆*E* value of 817.19 cm^−1^, which is marginally higher than the actual energy level difference in Er^3+^. This discrepancy may arise from the self-absorption of UCL by the lattice or from variations in the laser power during the experiments.

The relative and absolute sensitivities, key metrics for assessing temperature sensors, are calculated using the following equations [31,32,33]:

(5) $$s_{A}=|\frac{dFIR}{dT}|=|A*\exp\left(-\frac{∆E}{K_{B}T}\right)\left(\frac{∆E}{K_{B}T^{2}}\right)|$$

(6) $$s_{R}=\frac{1}{FIR}|\frac{dFIR}{dT}|=|\frac{∆E}{K_{B}T^{2}}|$$

Figure 6b,c illustrate the changes in the absolute and relative sensitivities at different temperatures. As the temperature rises from 353 to 453 K, *S_A_* gradually increases, reaching a maximum value of 0.0060 K^−1^ at 453 K. Moreover, *S_R_* reaches its peak value of 0.0094 K^−1^ at 353 K.

#### 5.1.2. Dual-Mode Sensing Characteristics at Low-Temperatures

**Figure 7:**
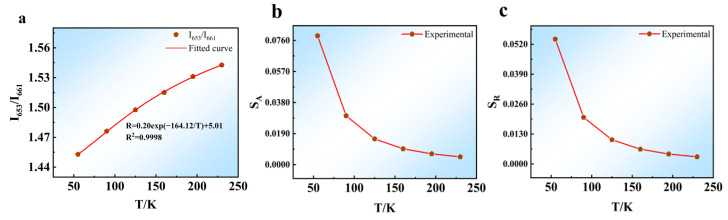
Temperature-sensing characteristics of the red emission. (**a**) FIR and *T* fitting, (**b**) *S_A_* vs. *T* relationship diagram, and (**c**) *S_R_* vs. *T* relationship diagram.

As shown in Figure 7a, the upconversion luminescence for the red emission demonstrates the dependence of the *I*_653_/*I*_661_ ratio at low temperatures. Through fitting the experimental data, we obtained ∆*E*/*k* = 164.12 K^−1^, which yields an energy level gap ∆*E* of 113.99 cm^−1^ between ^4^F_9/2(1)_ and 4F_9/2(2)_. Similarly, Equations (5) and (6) are used to calculate the absolute and relative sensitivities of the red emission under low-temperature conditions, as shown in Figure 7b,c. At 55 K, *S_A_* reaches a maximum value of 0.0788 K^−1^, and *S_R_* is 0.0543 K^−1^.

#### 5.1.3. Comparison of the High and Low-Temperature Conditions in Dual Mode

Based on the above data, the green emission was determined experimentally, and ∆*E* was 817.19 cm^−1^, which is close to the theoretical value of 800 cm^−1^ at high temperatures (353–453 K). At low temperatures, the particle population at the ^2^H_11/2_ level is relatively small, with most particles populating the lower ^4^S_3/2_ level. This results in prominent luminescence at 541 nm but not at 520 nm. In contrast, the experimental value ∆*E* = 113.99 cm^−1^ of red emission at low temperatures (55–230 K) is more consistent with the theoretical value of 100 cm^−1^. This aligns with the conclusion derived from Equation (3). In addition, the energy gap between the ^4^F_9/2(1)_ and ^4^F_9/2(2)_ levels is relatively small. At high temperatures, the spectral lines at these two levels tend to overlap to some extent. This overlap contributes to the deviation of the fitting value from the theoretical value at high temperatures. In contrast, under high-temperature conditions, the particle population in the ^4^F_9/2_ level reaches a saturated state, resulting in minimal changes in the upconversion emission intensity.

Based on the temperature-sensing performance of thermally coupled levels of Er^3+^, the experimental data of the system were compared with those reported in the literature and shown in Table 1. It was found that the *S_A_* and *S_R_* values of this sample were better than those reported in most literature. Therefore, the material can be used as an FIR-based optical temperature measurement material.

## 6. Conclusions

In this study, NaYF_4_:20%Yb^3+^/2%Er^3+^ upconversion microcrystals with a high upconversion emission intensity were synthesized using hydrothermal methods, and their temperature-dependent upconversion luminescence behavior was explored. We found that both the frequency- and time-domain upconversion emission spectra of these crystals depend strongly on temperature. A novel dual-mode luminescence thermometry strategy was developed, using the green (^2^H_11/2_→^4^I_15/2_ and ^4^S_3/2_→^4^I_15/2_) and red (^4^F_9/2_→^4^I_15/2_) upconversion emission intensity ratios and extending the temperature measurement range across both high and low temperatures. We also investigated the thermal coupling levels of Er^3+^ ions, particularly ^2^H_11/2_, ^4^S_3/2_, and the ^4^F_9/2(1)_ and ^4^F_9/2(2)_ pair, and we compared their temperature-sensing capabilities and energy level differences. The results showed that the energy level differences for green emission at high temperatures and red emission at low temperatures were in close agreement with the theoretical values, demonstrating the practicality and effectiveness of these upconversion microcrystals in temperature measurement applications. This shows that the system has a certain application potential in multi-mode temperature sensing.

## Figures and Tables

**Table 1:** Comparison of temperature-sensing performance based on Er^3+^ thermal coupling level.

Phosphors	Transitions	*T* (K)	*S_A_* (K^−1^)	*S_R_* (K^−1^)	Refs.
Y_2_O_3_:Er^3+^	^2^H_11/2_→^4^I_15/2_/^4^S_3/2_→^4^I_15/2_	298–393	0.0027	0.0154	[34]
NaYF_4_:Yb^3+^/Er^3+^	^2^H_11/2_→^4^I_15/2_/^4^S_3/2_→^4^I_15/2_	223–403	0.0037	-	[35]
YMoO_4_:Yb^3+^/Er^3+^	^2^H_11/2_→^4^I_15/2_/^4^S_3/2_→^4^I_15/2_	300–523	0.0035	-	[36]
NaYF_4_:Yb^3+^/Er^3+^	^2^H_11/2_→^4^I_15/2_/^4^S_3/2_→^4^I_15/2_	50–500	-	0.012	[37]
NaScF_4_:Yb^3+^/Er^3+^	^2^H_11/2_→^4^I_15/2_/^4^S_3/2_→^4^I_15/2_	298–573	0.0033	0.0041	[38]
NaYF_4_:Yb^3+^/Er^3+^	^2^H_11/2_→^4^I_15/2_/^4^S_3/2_→^4^I_15/2_	353–453	0.0060	0.0094	This work
NaYF_4_:Yb^3+^/Er^3+^	^4^F_9/2(1)_→^4^I_15/2_/^4^F_9/2(2)_→^4^I_15/2_	55–230	0.0788	0.0543	This work

## Data Availability

Data are contained within the article.
